# A cohort study of the relationship between anaemia, mean corpuscular volume and mortality among a CKD population in South Africa

**DOI:** 10.4314/ahs.v21i4.33

**Published:** 2021-12

**Authors:** Aishatu Nalado, Bala Waziri, Gbenga Olorunfemi, Johnny Mahlangu, Graham Paget, Raquel Duarte, Saraladevi Naicker

**Affiliations:** 1 Department of Internal Medicine, College of Health Sciences, Bayero University, Kano, Nigeria; 2 Department of Internal Medicine, School of Clinical Medicine, Faculty of Health Science, University of the Witwatersrand, Johannesburg, South Africa; 3 Department of Medicine, Ibrahim Badamasi Babangida Specialist Hospital, Minna, Nigeria; 4 Division of Epidemiology and Biostatistics, School of Public Health, University of the Witwatersrand, Johannesburg, South Africa; 5 School of Pathology, Faculty of Health Sciences, University of the Witwatersrand, Johannesburg, South Africa

**Keywords:** Chronic kidney disease, mortality anaemia, outcomes, survival

## Abstract

**Background:**

The burden of chronic kidney disease is increasing globally and prompt identification, coupled with improved management of CKD patients have increased the population of pre-dialysis patients. We, therefore, aimed to evaluate the predictors of survival among pre-dialysis CKD patients in South Africa.

**Methods:**

We conducted a cohort study of 256 consecutive consenting Black non-dialysis requiring CKD patients attending the renal outpatient clinic of a tertiary Hospital in South Africa from 1st June 2016 to 1st December 2016. Socio-demographic and clinical information of the participants were obtained. Descriptive statistics, Kaplan-Meier curves and Cox proportional hazard regression analyses were conducted to evaluate factors affecting the survival of the participants.

**Results:**

The mean age of the participants was 52.8±14.3 years and 48.0% were females, 52% were males. The death rate increased with worsening haemoglobin level from 0.96 among patients with mild anaemia to 4.29 per 100-person years among patients with severe anaemia. Anaemic patients with GFR < 30mls/min had significantly increased risk of death (HR 11.51, 95% CI 1.62–78.32, P < 0.001).

**Conclusion:**

Mortality in pre-dialysis CKD patients was associated with anaemia and hyperphosphatemia. Clinical interventions targeted at preventing these conditions may improve outcomes among this group of CKD patients.

## Introduction

Chronic kidney disease (CKD) is an increasing global public health problem, and Africa remains one of the worst affected regions^1^. The rising trends in the number of CKD patients in Africa may be due to increased awareness, early identification, and improvement in the management of CKD patients, hence there is a need to identify potentially modifiable risk factors of mortality in this growing number of pre-dialysis patients.

The survival of non-dialysis CKD patients is a complex interplay of environment, management and co-morbidities^2^, among others. Anaemia is a major comorbidity among CKD patients. However, the degree to which anaemia impacts on mortality rates among nondialysis CKD patients has not been well characterised in South Africa^2^, ^3^. Other risk factors influencing mortality in pre dialysis CKD patients include diabetic nephropathy, polycystic kidney disease, evidence of interstitial fibrosis 4, 5, 6, male gender, black race, specific genotypes, proteinuria, poor glycaemic control/ diabetes, higher blood pressure, obesity, lower serum high density lipo-protein (HDL) cholesterol, and smoking^7^–^9^. In addition, the high cost of treatment and level of renal function can also be associated with increased risk of morbidity and mortality among CKD patients. Furthermore, the outcomes and survival of non-dialysis CKD patients in our environment is expected to be different from that in high-income countries because of differences in provision of optimum care, socio-demographic characteristics, genetic factors and other associated comorbidities.

While patients attending renal replacement programmes are expected to have better outcomes, the efficacy of the chronic pre-dialysis programme is not yet well characterised in our environment. Thus, knowledge of the survival rates and factors affecting outcomes among non-dialysis CKD patients can be used to assess the impact of current management protocols in our environment. Furthermore, special attention can be devoted to preventing or improving identified major factors influencing survival in our environment. South Africa is a multi-ethnic country, and socio-economic status is largely related to ethnicity. Risk factors predicting mortality and morbidity among dialysis CKD patients, and survival outcomes have been well studied previously, but they are less well-defined in the pre-dialysis CKD patients. Renal replacement therapy, though available, is accessible to only a few South African CKD patients; there is a policy that only patients attending state dialysis facilities can access free dialysis in South Africa, and as only a limited number of patients qualify for the state dialysis programmes, many patients die before they are able to access dialysis. Long term survival in predialysis patients with CKD is low and this can be attributable to various medical and social conditions^7^.

Thus, this study was conducted to evaluate the predictors of survival among black non-dialysis CKD patients in a hospital in South Africa and to generate data among this group of patients in order to guide in their management and improve in their outcome.

## Methods

This study was a cohort study of 256 Black consenting pre-dialysis CKD patients attending the renal outpatient clinic of a Hospital in South Africa, who were recruited from 1 June to 1 December 2016, and were then followed up till 1 December 2017. Following approval from the Human Research Ethics Committee of the University(Certificate number supplied to the editor), participants' socio-demographic and clinical information was obtained using a self-administered proforma. Participants were then followed up monthly, and some patients were followed up 3 monthly depending on their clinical conditions during clinic visits and those that did not attend clinic were contacted telephonically to elicit information about their survival. When necessary, patients' relatives were also contacted to ascertain if death had occurred and to further ascertain when it actually occurred. Hospital mortality records were reviewed to ascertain details of those that died in the hospital. Participants who were older than 18 years were included in the study. Exclusion criteria included patients with active infecion, inflammation, malignancy, and patients with a 3-month history of blood transfusion prior to enrolment; those on immunosuppressants were also excluded from the study. Data collected included demographic characteristics, blood pressure measurements, and medication history. The primary outcome of this study was death; other events such as commencement of dialysis, transplantation, and being alive at the end of the study were censored.

Biochemical measurements (urea, creatinine, calcium, phosphate), were measured using an autoanalyzer (Siemens Diagnostics, Tarry Town, NY Inc.). Haemoglobin was measured using a Siemens ADVIA 120, 2\20 and Technion H 3 RTX and RTCautoanalyzer (Siemens Medical Solutions Diagnostics, Tarrytown, NY)

## Statistical analysis

Continuous variables are presented as means± standard deviations or medians (interquartile ranges) depending on the distribution of the parameter. Comparison of baseline parameters between patients who were alive and dead was carried using Chi-square for categorical variables, and Student's t-test and Wilcoxon rank-sum test for continuous variables. Participants were further categorized into four categories based on the severity (normal, mild, moderate, severe) of anaemia according to the World Health Organisation criteria. The analysis of variance (ANOVA) and Kruskal Wallis tests were used as appropriate in comparing baseline characteristics among the four categories of participants. A Post-hoc Bonferroni test was conducted as appropriate.

Proportional hazards assumption was checked using both graphical visualization and Schoenfeld residuals test. The association between anaemia status and mortality was described using Kaplan Meier survival curves and the log-rank test was conducted to evaluate the associations. Time to death was the time variable.

Events other than death such as lost to follow up, commencement of dialysis, kidney transplantation and being alive at the end of the study were right censored. Univariable Cox hazard proportional analysis was conducted between each exploratory variable and the primary outcome (death). Multivariable Cox hazard proportional analysis was then conducted using a stepwise backward elimination approach to adjust for the confounding variables in the relationship between anaemia and mortality. Independent variables with P-value <0.2 were added into the model. The independent variables were Hb, serum albumin and phosphate levels, GFR, (which was determined using the formulae Chronic Kidney Disease Epidemiology Collaboration (CKDEPI) equation for GFR(9), and systolic blood pressure. However, variables (such as age, gender and CRP) that were known to be biologically associated with mortality were defined a priori and included in the multivariable model regardless of their univariable P value. The association between anaemia and mortality was evaluated by building three different models (A, B, C). Model A included anaemia as dichotomous (either present/ absent); Model B included the four categories of anaemia (normal, mild, moderate, severe) and in model C, the variable ‘haemoglobin’ was included as a continuous variable. Hazard ratios (with 95% confidence interval) were then generated for the variables.

The study participants were dichotomised into low versus high mean corpuscular volume (MCV) categories using the median MCV of 88.0 fl. We further explored the predictors of mortality by including an interaction term in the multivariable model between anaemia and GFR. Statistically, significant level was set at 95% confidence interval (P-value<0.05). All analyses were performed using Stata version 13 (STATA Corp., TX, and USA) statistical software.

## Results

Of the 256 study participants, 52.0% were males while 48.0% were females. The mean age was 52.8±14.3 years. During a median follow up time of 15 months, 40 (15.6%) of the participants had died, a mortality rate of 1.12 per 100 person- years. The death rate increased with decreasing haemoglobin levels; thus, the mortality rate among participants with severe anaemia (4.29 per 100 person-years) was about four-fold the mortality rate among participants with mild anaemia (0.96 per 100-person years). The overall prevalence of anaemia was 47.6%. The mean levels of albumin and Hb were lower among the participants that died, while median serum ferritin and mean serum phosphate levels were higher among those that died in comparison to those that survived (Table 1).

**Table 1:** Baseline characteristics of the study population by mortality

Variable	All (n=256)	Alive (n=216)	Dead (n=40)	P-value
Age (years)	52.8±14.3	52.7±14.6	53.4±12.9	0.79
Gender n (%) Male Female	133 (52.0) 123 (48.0)	110 (50.9) 106 (49.1)	23 (57.5) 17 (42.5)	0.27
Hb (g/dl)	12.2± 2.7	12.6± 2.5	9.7± 2.7	<0.0001
Median MCV (fl)	88 (83–92)	88 (84–93)	85 (82–89)	0.009
Ferritin ng/mL	104 (57–198)	99 (56–179)	211 (100–394)	<0.0001
BMI (kg/m^2^)	29.9±6.4	30.0±6.5	29.1± 6.5	0.43
Systolic BP (mmHg)	143.7± 22.9	142.9± 21.7	148.1± 22.9	0.20
Diastolic BP (mmHg)	81.7± 15.6	81.6± 17.3	82.4± 15.6	0.78
Albumin (g/L)	39.5± 5.4	39.8±5.2	37.4± 5.5	0.008
T.Chol (mmol/L)	4.3± 1.6	4.3±1.6	4.2±1.8	0.63
HDL (mmol/L)	1.2±0.4	1.2±0.4	1.1±0.4	0.51
LDL(mmol/L)	2.6± 1.4	2.7±1.4	2.6±1.4	0.22
TG (mmol/L)	1.6±1.0	1.6±1.0	1.4±1.0	0.36
Phosphate (mmol/L)	1.23±0 .56	1.10±0.30	1.93±0.97	<0.0001
Diabetes mellitus n (%)	87 (34.0)	73 (33.8)	14 (35.0)	0.06
Stages of CKD n (%)				
Stage 1	16 (6.3)	15 (6.9)	1 (2.5)	<0.001^a^
Stage 2	30 (11.2)	28 (13.0)	2 (5.0)	
Stage 3a	33 (12.9)	32 (14.8)	1 (2.5)	
Stage 3b	58 (22.7)	57 (26.4)	1 (2.5)	
Stage 4	60 (23.4)	55 (25.5)	5 (12.5)	
Stage 5	59 (23.0)	29 (13.4)	30 (75.0)	
CRP mg/L	20.1±30.6	30.0±55.6	12.9± 8.5	0.002
Median GFR (mls/min)	32 (16–52)	37 (21–55)	8 (4–12)	<0.0001

T. Chol= total cholesterol, BP= blood pressure, LDL=low density lipoprotein, HDL=high density, MCV= mean corpuscular volume

a p value for comparison of stages of CKD between patients who were alive and dead.

Anaemic patients had lower mean serum albumin levels and lower median GFR, with higher mean serum phosphate than the non-anaemic patients (Table 2).

**Table 2:** Baseline characteristics of the study population by anaemia status

Variable	All (n=256)	Anaemic (n=122)	Non -anaemic (n=134)	P-value
Age (years)	52.8±14.3	52.4±13.9	53.2±14.7	0.68
Gender n (%) Male Female	133 (52.0) 123 (48.0)	59 (48.4) 63 (51.6)	74 (55.2) 60 (44.8)	0.27
Hb (g/dl)	12.17± 2.74	9.99± 1.99	14.15± 1.56	<0.0001
MCV (fl)	87.6±6.2	86.6±6.5	88.6±5.6	0.007
BMI (kg/m^2^)	29.89±6.38	29.41±6.85	30.33± 5.905	0.25
Systolic BP (mmHg)	143.74± 22.91	141.93± 24.80	145.40± 21.00	0.23
Diastolic BP (mmHg)	81.75± 15.56	80.68± 16.31	82.73± 14.81	0.29
Albumin (g/L)	39.45± 5.36	37.88±5.56	40.89± 4.750	<0.0001
T.Chol (mmol/L)	4.31± 1.63	4.16±1.45	4.46±1.75	0.14
HDL (mmol/L)	1.16±0.41	1.13±0.43	1.18±0.38	0.34
LDL(mmol/L)	2.64± 1.43	2.53±1.23	2.74±1.60	0.22
TG (mmol/L)	1.56±0.97	1.45±0.96	1.66±0.97	0.09
Phosphate (mmol/L)	1.23±0.56	1.46±0.70	1.02±0.25	<0.0001
Diabetes mellitus n (%)	87 (34.0)	52 (20.5)	35 (26.1)	0.005
Stages of CKD, n (%)				
Stage 1	16 (6.3)	7 (5.7)	9 (6.7)	<0.0001
Stage 2	30 (11.7)	8 (6.6)	22 (16.4)	
Stage 3a	32 (12.5)	7 (5.7)	25 (18.7)	
Stage 3b	58 (22.7)	15 (12.3)	43 (32.1)	
Stage 4	59 (23.1)	31(25.4)	28 (20.9)	
Stage 5	59 (23.1)	54 (44.3)	5 (3.7)	
CRPmg/L	20.1±30.6	30.0±55.6	12.9± 8.5	0.002
Median GFR (mls/min)	32 (16–52)	19 (8–38)	42 (30–58)	<0.0001

T. Chol: total cholesterol, BP:blood pressure, LDL:low density lipoprotein, HDL:high density lipoprotein, TG:triglyceride, Hb: haemoglobin, GFR: glomerular filtration rate, MCV: mean corpuscular volume. a: p value for comparison of stages of CKD between patients who were alive and dead.

Serum albumin levels decreased with worsening anaemia state (Table 3), while the mean phosphate level progressively increased with increasing severity of anaemia. Other parameters were comparable between the categories of anaemia.

**Table 3:** Baseline characteristics of the study participants by severity of anaemia

Variable	Normal (n= 47)	Mild anaemia (n=29)	Moderate anaemia (n=42)	Severe anaemia (n=38)	P value
Death rate, per 100-person years, n (rate)	7 (0.31)	4 (0.36)	12 (1.32)	17 (2.66)	
Age (years)	52.9± 14.4	55.6±14.1	52.3±15.4	50.8±13.1	0.60
Hb (g/dl)	14.0± 1.5	11.6± 0.3	10.1± 0.6	7.5±1.2	<0.0001
BMI(kg/m2)	30.1±5.8	29.8±6.1	30.6± 6.7	28.9±8.2	0.65
Systolic BP (mmHg)	145.3± 21.3	146.0± 25.2	140.8± 27.5	139.2±21.5	0.38
Diastolic BP (mmHg)	82.9± 15.5	84.1 ± 15.8	78.5± 15.1	78.9±15.8	0.20
Albumin (g/L)	40.8± 4.8	39.5±5.0	38.7± 5.1	34.9±5.6	<0.0001
T.Chol (mmol/L)	4.41± 1.74	4.46±1.00	4.14±1.42	4.0±1.74	0.49
HDL (mmol/L)	1.19±0.42	1.21±0.28	1.10±0.35	1.04±0.46	0.13
LDL (mmol/L)	2.68± 1.57	2.76±1.00	2.54±1.15	2.51±1.51	0.83
TG (mmol/L)	1.67±0.96	1.40±0.85	1.36±0.70	1.51±1.29	0.21
Phosphate (mmol/L)	1.01±0.21	1.15±0.21	1.31±0.49	2.03±0.90	<0.001
Diabetes	44 (29.9)	8 (27.6)	19 (45.2)	16 (42.1)	0.15
GFR (mls/min)	40 (27–58)	28 (16–57)	21(10–35)	7 (3–19)	0.0001

T. Chol= total cholesterol, BP= blood pressure, LDL=low density lipoprotein, HDL=high density lipoprotein, TG=triglyceride, Hb= haemoglobin, GFR=glomerular filtration rate

**Figure 1:**
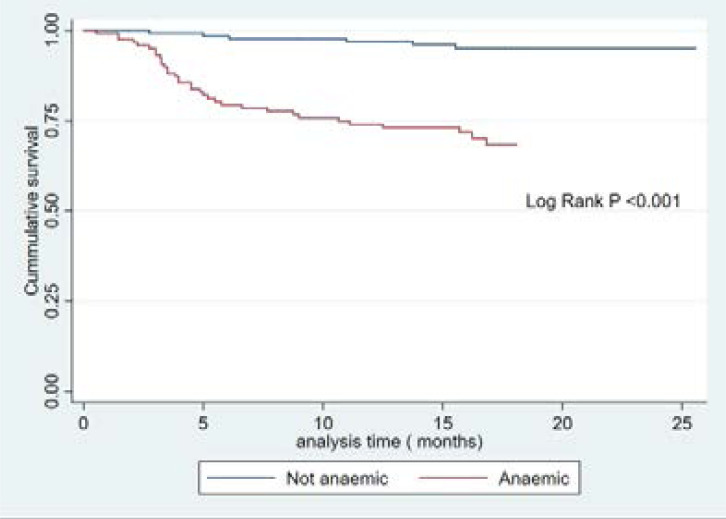
Kaplan Meier survival curve comparing anaemic to non-anaemic patients

**Figure 2:**
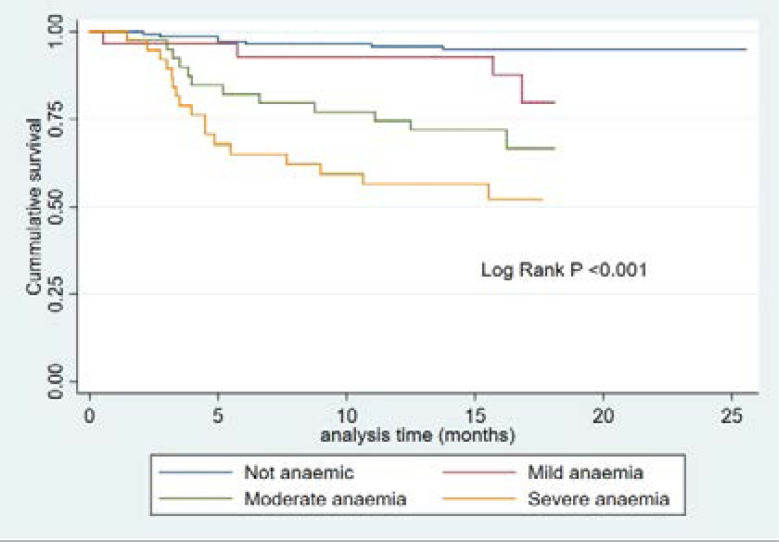
Kaplan Meier survival curve of the participants stratified by categories of anaemia severity

Figures 1 and 2 show the Kaplan Meier analysis of the association between anaemia and mortality in patients stratified by their anaemia status and the severity of anaemia, respectively. Anaemic patients were at increased risk of death in comparison to non-anaemic patients with increasing time of follow-up (log rank p <0.001). Thus, about 75% of the anaemic participants survived beyond 12 months of follow-up, while about 90% of the non-anaemic participants survived beyond 12 months of follow-up. Similarly, patients with moderate and severe anaemia have the worst survival outcomes (log rank p <0.001).

In univariable analysis, anaemia was strongly associated with an increased hazard of death (unadjusted hazard ratio (HR) = 7.53, 95% (CI): 3.16–17.95), P-value < 0.001). After adjusting for confounders, the hazard of death among anaemic participants was 4.7-fold higher than the hazard of death among non-anaemic participants (Adj HR: 4.68; 95 % CI:1.64–13.34, P=0.004). This association was further explored by stratifying participants based on the severity of anaemia. In comparison to non-anaemic participants, the adjusted HRs increases with severity (for moderate anaemia adj HR 7.20; 95% CI, 2.83–18.20, P=0.007) and for severe anaemia (adj HR, 12.68; 95% CI, 5.24–30.65, P=0.005). Considering Hb level as a continuous variable, higher haemoglobin levels were associated with decreased hazard of death for every g/dl increase in Hb levels (HR 0.78; 95%CI, 0.67–0.91, P=0.002) (Table 4).

**Table 4:** Unadjusted and adjusted hazard ratios for all-cause mortality in the study participants

Methods	HR (95%CI)	P value	Adjusted HR (95%CI)	P-value
Model A				
Non-anaemic	1.00 (reference)		1.00 (reference)	
Anaemic	7.53 (3.16–17.95)	<0.001	4.73 (1.66–13.50)	0.004
Model B				
No anaemia	1.00 (reference)		1.00 (reference)	
Mild anaemia	3.02 (0.88–10.31)	0.08	2.59 (0.73–9.30)	0.12
Moderate anaemia	7.20 (2.83–18.20)	<0.001	5.20 (1.77–15.25)	0.003
Severe anaemia	12.68 (5.24–30.65)	<0.001	5.84 (1.88–18.13)	0.002
Model C Hb as continuous variable				
Hb	0.69 (0.62–0.78)	<0.001	0.76(0.65–0.88)	0.001

Models A-C adjusted for age, gender, systolic blood pressure, diabetic status, glomerular filtration rate, serum phosphate and CRP. Hb= haemoglobin, HR=Hazard ratio, CI=confidence interval.

**Figure 3:**
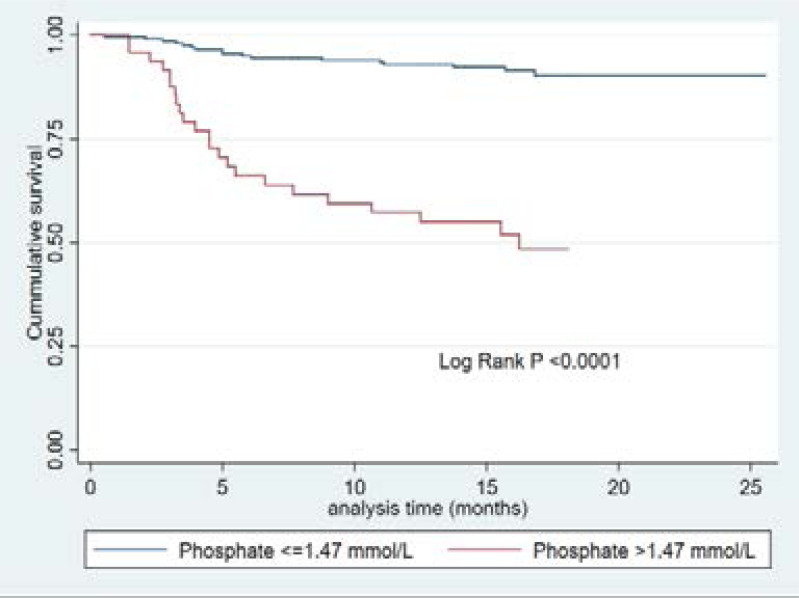
Kaplan Meier survival curve for phosphate levels.

Another independent predictor of mortality was hyperphosphatemia (defined as phosphate level >1.47 mmol/L). As shown in Figure 3, hyperphosphatemia was associated with decreased survival, log rank p <0.0001. The association between hyperphosphatemia and mortality persisted after adjusting for potential confounders (HR, 2.60; 95%CI, 1.09–6.20, P =0.02).

The median MCV was 88.0 fl, IQR 83.4 – 91.6. Based on the median MCV (normal range MCV is 80–95fl/red cells in an adult), the study participants were categorized into low MCV (<88.0 fl) group and high MCV (≥88.0 fl) group. On univariable analysis, compared to low MCV group, patients in the high MCV group had reduced risk of mortality (HR, 0.52; 95% CI, 0.27–0.99; P=0.046). This association was not statistically significant in the multivariable analysis; (adjusted HR, 0.79; 95% CI, 0.40–1.57; P=0.51).

We further assessed for an interaction between Hb and GFR by dichotomizing GFR as < 30 mls/min versus ≥30 mls/min. An interaction was found between anaemia and lower GFR < 30mls/min as shown by statistical significance of the cross product of Hb and GFR < 30mls/min in the fully adjusted model. Anaemic patients with GFR < 30mls/min had significantly increased risk of death (HR 11.51, 95% CI 1.62–78.32, P < 0.001).

The Schoenfeld test for the final models shows no evidence of violation of the proportional hazards model (global test p value of 0.48).

## Discussion

This study sought to evaluate the factors affecting mortality among Black pre-dialysis CKD patients in a South African Hospital and has identified clinical and biochemical predictors of mortality in these patients. The study showed that after a follow- up period of eighteen months, about 15.6% died at a rate of 1.12 per 100 persons- years. The death rate quadrupled among participants with severe anaemia (4.96 per 100 personyears) as compared to the death rate among participants with mild anaemia (0.96 per100- person- year); these results are lower than the findings of 57.9% in Ethiopia^10^, and 67.4% in Europe^11^. The explanation for the lower mortality rate in our study could be related to the varying socioeconomic status of the different studies. Furthermore, these previous studies only followed-up their participants for 90 days as opposed to our eighteen months follow up period. Although previous studies may not have followed up their participants long enough to identify higher mortality rates, the mortality rate in our study was still lower. In addition, while our study participants were pre-dialysis patients, the previous researchers studied patients that were already on haemodialysis. It is to be expected that the cohort of CKD patients on haemodialysis would have higher mortality rates in comparison to pre-dialysis patients. However, our cohort of predialysis patients who were monitored for longer periods still had lower mortality rates than those reported in previous studies^12^–^15^, and our cohort was younger, and had lesser comorbidities than those of other researchers, which could explain the lower mortality^12^–^15^. Our results need to be explored with a larger sample size. Our finding also suggests that there may be other individual, environmental or genetic factors that may influence survival of pre-dialysis CKD patients. Anaemia can be a major indicator of mortality, poor health, and low socio-economic status in many settings^12^. Our study showed that the cumulative 1-year survival rate among anaemic participants was 75% while the 1-year survival rate among non-anaemic participants was higher (about 90%). Furthermore, we showed that the estimated risk of death from anaemic as compared to non-anaemic CKD patients was about 5-fold. (HR= 4.68 95% CI 1.64–13.34). Our findings are consistent with other studies on CKD patients, as anaemia is strongly associated with adverse clinical outcomes including mortality^8^, ^13^, ^14^. One of the reasons for increased risk of death from anaemia in CKD patients may be due to haemodynamic decompensations, and worsening hypoxia. These decompensations will further stress left ventricular function and worsen heart failure in patients with heart failure. Furthermore, anaemia will cause further changes in the anatomy of the left ventricle, and such changes may increase the risk of death^15^.

We further assessed interaction between anaemia and GFR by dichotomizing GFR as <30mls/min versus >30mls/min; an interaction was found between anaemic patients with GFR <30mls/min, as there was an increased risk of death in this group (HR 11.5 95% CI 1.62–78.32, p<0.001). This finding of a relationship between kidney function, anaemia and mortality are consistent with prior reports^8^, ^16^, ^17^. However, Culleton et al. showed that the impact of anaemia, hospitalization and mortality were greatest among patients with normal kidney function. The difference in the findings of Culleton et al could be explained by the differences in the ages of their study population, and prevalence of associated co-morbidities^13^. Although the mechanism by which CKD potentiates risk of death is unknown, possible explanations are coexistence with other cardiovascular risk factors like anaemia, diabetes, patients with renal disease receiving efficacious, but potentially toxic therapies, and associated vascular disease (atherosclerosis); these risk factors might contribute directly to mortality in these patients^14^, ^18^–^20^.

We found that higher levels of phosphate were associated with increased mortality in pre- dialysis CKD patients. Our finding is consistent with findings by Kestenbaum et al, in the Framingham offspring study^21^,^22^. Similarly, Voormolen et al conducted a prospective study in pre-dialysis CKD patients with GFR <20mls/min and found an association of increased phosphate with progression of CKD and mortality^23^, although our cut- off value for phosphate was different. Contrary to our findings, while Menon et al, did not find an association between phosphate levels and mortality, Block et al, showed that lower phosphate levels were associated with increased mortality^24^, ^25^. The possible explanation for the association between hyperphosphatemia and increased mortality in our study may be related to a pathological link between increased phosphate levels and vascular calcifications, but this finding needs to be further explored^26^, ^27^. Furthermore, high phosphate levels have also been associated with left ventricular hypertrophy^28^,^29^ that could exacerbate the prevalence of cardiovascular diseases and death among CKD patients^30^.

In this present study, there was a lack of significant association between MCV and mortality in the adjusted model. This finding is consistent with findings of Peng et al^31^, in incident peritoneal dialysis patients, MCV was not statistically significantly associated with mortality in the fully adjusted model, and is consistent with findings of Chen et al^32^ among haemodialysis patients, where the two year survival rate was significantly lower in the high red cell distribution width group, Balta et al^33^, found lower death rate among patients with higher MCV, though his cohort were patients with myocardial infarction. This finding is in contrast to findings by Hsieh et al among stage 3–5 CKD patients^34^, Sun et al among peritoneal dialysis patients in Korea, Brain et al^35^ and Vashistha et al^36^ among haemodialysis patients. The differences in results between our study and other studies may partly be explained by differences in patient characteristics, race, and diverse healthcare delivery systems. Though MCV has been associated with mortality in clinical settings^37^–^39^, the association of MCV with mortality in CKD patients' needs to be further explored with a larger sample size, in a prospective multicentre study, to confirm the association.

The recommended phosphate target levels among CKD patients, according to the KDOQI guidelines, was developed from evidence and results obtained from studies conducted in haemodialysis patients^40^. Although, our study tends to agre with the phosphate level targets of KDOQI, we surmise that more studies like ours are necessary among pre-dialysis CKD patients so as to be able to make recommendations among the growing pre-dialysis CKD population. It may therefore not be appropriate to extrapolate the recommendations obtained from patients on haemodialysis to pre-dialysis patients.

The strength of our study is in the prospective cohort design. This helps us to build in length of follow up (of up to 18 months) into our conclusions. However, this study should be interpreted in the light of its limitations. Although our sample size was reasonably large (256 participants), since we studied multiple risk factors, other risk factors that were currently not statistically associated with mortality may become associated in a larger sample size. The telephone conversation to ascertain the date of mortality of some of the participants may potentially not be an accurate method. But we reasonably believe the patients' relatives that told us of the date of death since they were either next of kin and/or care givers while the patients were alive. During recruitment we also counselled the participants and care givers about the end points of the study.

Despite these limitations, our study has important strengths including its prospective design, serial measurements of variables like haemoglobin and creatinine over a period of time; in addition, our patients were free from active infections, inflammation, and malignancy. In conclusion, our findings suggest that anaemia, worsening kidney function, and hyperphosphatemia, are associated with increased mortality in non- dialysis black CKD patients. Thus, extra care should be taken to identify and promptly treat anaemia and hyperphosphatemia among the pre-dialysis CKD patients.

## References

[1] Muntner P, Coresh J, Klag MJ (2002). History of myocardial infarction and stroke among incident end-stage renal disease cases and population-based controls: an analysis of shared risk factors. American Journal of Kidney Diseases: The official Journal of the National Kidney Foundation.

[2] Horl WH (2013). Anaemia management and mortality risk in chronic kidney disease. Nature Reviews Nephrology.

[3] Pedro L, Neves EM, Baptista Alexandre (2007). Anaemia and Interleukin-6 Are Associated with Faster Progression to End- Stage Renal Disease. Dialysis and Transplantation.

[4] Kussman MJ, Goldstein H, Gleason RE (1976). The clinical course of diabetic nephropathy. JAMA.

[5] Klahr S, Breyer JA, Beck GJ, Dennis VW, Hartman JA, Roth D (1995). Dietary protein restriction, blood pressure control, and the progression of polycystic kidney disease. Modification of Diet in Renal Disease Study Group. Journal of the American Society of Nephrology.

[6] Nath KA (1992). Tubulointerstitial changes as a major determinant in the progression of renal damage. American Journal of Kidney Diseases: The Official Journal of The National Kidney Foundation.

[7] Barsoum RS (2006). Chronic kidney disease in the developing world. The New England journal of medicine.

[8] Vlagopoulos PT, Tighiouart H, Weiner DE, Griffith J, Pettitt D, Salem DN (2005). Anemia as a risk factor for cardiovascular disease and all-cause mortality in diabetes: the impact of chronic kidney disease. Journal of the American Society of Nephrology.

[9] van den Brand GAJvB AJGJan, Willems Hans L (2011). Introduction of the CKD-EPI equation to estimate glomerular filtration rate in a Caucasian population. Nephrology Dialysis Transplantation.

[10] Shibiru T, Gudina EK, Habte B, Deribew A, Agonafer T (2013). Survival patterns of patients on maintenance hemodialysis for end stage renal disease in Ethiopia: summary of 91 cases. BMC Nephrology.

[11] van Dijk PC, Jager KJ, de Charro F, Collart F, Cornet R, Dekker FW (2001). Renal replacement therapy in Europe: the results of a collaborative effort by the ERA-EDTA registry and six national or regional registries. Nephrology, dialysis, transplantation: official publication of the European Dialysis and Transplant Association - European Renal Association.

[12] Righetti AA, Koua AYG, Adiossan LG, Glinz D, Hurrell RF, N'Goran EK (2012). Etiology of Anemia Among Infants, School-Aged Children, and Young Non-Pregnant Women in Different Settings of South-Central Côte d'Ivoire. The American Journal of Tropical Medicine and Hygiene.

[13] Culleton BF, Manns BJ, Zhang J, Tonelli M, Klarenbach S, Hemmelgarn BR (2006). Impact of anemia on hospitalization and mortality in older adults. Blood.

[14] Zakai NA, Katz R, Hirsch C, Shlipak MG, Chaves PH, Newman AB (2005). A prospective study of anemia status, hemoglobin concentration, and mortality in an elderly cohort: the Cardiovascular Health Study. Archives of internal medicine.

[15] Del Fabbro P, Luthi JC, Carrera E, Michel P, Burnier M, Burnand B (2010). Anemia and chronic kidney disease are potential risk factors for mortality in stroke patients: a historic cohort study. BMC Nephrology.

[16] Sarnak MJ, Tighiouart H, Manjunath G, MacLeod B, Griffith J, Salem D (2002). Anemia as a risk factor for cardiovascular disease in The Atherosclerosis Risk in Communities (ARIC) study. Jornal of the American College Cardiol.

[17] Abramson JL, Jurkovitz CT, Vaccarino V, Weintraub WS, McClellan W (2003). Chronic kidney disease, anemia, and incident stroke in a middle-aged, community-based population: the ARIC Study. Kidney international.

[18] Hannedouche T, Albouze G, Chauveau P, Lacour B, Jungers P (1993). Effects of blood pressure and antihypertensive treatment on progression of advanced chronic renal failure. American journal of kidney diseases: the official journal of the National Kidney Foundation.

[19] Shlipak MG, Heidenreich PA, Noguchi H, Chertow GM, Browner WS, McClellan MB (2002). Association of renal insufficiency with treatment and outcomes after myocardial infarction in elderly patients. Annals of internal medicine.

[20] Knight EL, Rimm EB, Pai JK, Rexrode KM, Cannuscio CC, Manson JE (2004). Kidney dysfunction, inflammation, and coronary events: a prospective study. Journal of the American Society of Nephrology.

[21] Dhingra R, Sullivan LM, Fox CS, Wang TJ, D'Agostino RB, Gaziano JM (2007). Relations of serum phosphorus and calcium levels to the incidence of cardiovascular disease in the community. Archives of internal medicine.

[22] Kestenbaum B, Sampson JN, Rudser KD, Patterson DJ, Seliger SL, Young B (2005). Serum phosphate levels and mortality risk among people with chronic kidney disease. Journal of the American Society of Nephrology.

[23] Voormolen N, Noordzij M, Grootendorst DC, Beetz I, Sijpkens YW, van Manen JG (2007). High plasma phosphate as a risk factor for decline in renal function and mortality in pre-dialysis patients. Nephrology, dialysis, transplantation: official publication of the European Dialysis and Transplant Association -European Renal Association.

[24] Menon V, Greene T, Pereira AA, Wang X, Beck GJ, Kusek JW (2005). Relationship of phosphorus and calcium-phosphorus product with mortality in CKD. American journal of kidney diseases: the official journal of the National Kidney Foundation.

[25] Block GA, Hulbert-Shearon TE, Levin NW, Port FK (1998). Association of serum phosphorus and calcium x phosphate product with mortality risk in chronic hemodialysis patients: a national study. American journal of kidney diseases: the official journal of the National Kidney Foundation.

[26] Ketteler M, Biggar PH (2009). Review article: Getting the balance right: assessing causes and extent of vascular calcification in chronic kidney disease. Nephrology (Carlton, Vic).

[27] Nikolov IG, Mozar A, Drueke TB, Massy ZA (2009). Impact of disturbances of calcium and phosphate metabolism on vascular calcification and clinical outcomes in patients with chronic kidney disease. Blood purification.

[28] Strozecki P, Adamowicz A, Nartowicz E, Odrowaz-Sypniewska G, Wlodarczyk Z, Manitius J (2001). Parathormon, calcium, phosphorus, and left ventricular structure and function in normotensive hemodialysis patients. Renal failure.

[29] Foley RN, Collins AJ, Herzog CA, Ishani A, Kalra PA (2009). Serum phosphorus levels associate with coronary atherosclerosis in young adults. Journal of the American Society of Nephrology.

[30] Shlipak MG, Katz R, Sarnak MJ, Fried LF, Newman AB, Stehman-Breen C (2006). Cystatin C and prognosis for cardiovascular and kidney outcomes in elderly persons without chronic kidney disease. Annals of internal medicine.

[31] Peng F, Li Z, Zhong Z, Luo Q, Guo Q, Huang F (2014). An increasing of red blood cell distribution width was associated with cardiovascular mortality in patients on peritoneal dialysis. International journal of cardiology.

[32] Chen X SB, Zou J (2016). The Prognostic Value of Red Blood Cell Distribution Width in Patients on Maintenance Hemodialysis. Blood purification.

[33] Balta S, Demirkol S, Aparci M, Arslan Z, Ozturk C (2015). Red Cell Distribution Width in Myocardial Infarction. Medical Principles and Practice.

[34] Hsieh YP, Chang CC, Kor CT, Yang Y, Wen YK, Chiu PF (2017). Mean Corpuscular Volume and Mortality in Patients with CKD. Clinical Journal of the American Society of Nephrology: CJASN.

[35] Michel BE (2011). The association of red blood cell parameters with mortality in a population of hemodialysis patients. Dialysis & Transplantation.

[36] Vashistha T, Streja E, Molnar MZ, Rhee CM, Moradi H, Soohoo M (2016). Red Cell Distribution Width and Mortality in Hemodialysis Patients. American journal of kidney diseases: the official journal of the National Kidney Foundation.

[37] Yoon HJ, Kim K, Nam YS, Yun JM, Park M (2016). Mean corpuscular volume levels and all-cause and liver cancer mortality. Clinical chemistry and laboratory medicine.

[38] Sičaja M, Pehar M, Đerek L, Starčević B, Vuletić V, Romić Ž (2013). Red blood cell distribution width as a prognostic marker of mortality in patients on chronic dialysis: a single center, prospective longitudinal study. Croatian Medical Journal.

[39] Bazick HS, Chang D, Mahadevappa K, Gibbons FK, Christopher KB (2011). Red Cell Distribution Width and all cause mortality in critically ill patients. Critical care medicine.

[40] (2003). K/DOQI clinical practice guidelines for bone metabolism and disease in chronic kidney disease. American journal of kidney diseases: the official journal of the National Kidney Foundation.

